# Malignant Wolffian adnexal tumor in the ovary: a case report and literature review

**DOI:** 10.3389/fonc.2025.1526030

**Published:** 2025-03-18

**Authors:** Cheng Chi, Guoliang Li, Zian Zheng, Xiangyu Wang, Xiangyu Liu

**Affiliations:** ^1^ Minjiang Road Community Health Service Center, Shinan District Medical Health Group, The Affiliated Hospital of Qingdao University, Qingdao, Shandong, China; ^2^ Department of Radiation Oncology, The Affiliated Hospital of Qingdao University, Qingdao, Shandong, China; ^3^ Medical Department, The Affiliated Hospital of Qingdao University, Qingdao, Shandong, China; ^4^ Department of Obstetrics and Gynecology, The Affiliated Hospital of Qingdao University, Qingdao, Shandong, China

**Keywords:** Wolffian adnexal tumor (WAT), female reproductive system, ovarian tumor, treatment, case report

## Abstract

**Background:**

Wolffian adnexal tumor (WAT) is a rare neoplasm originating from the remnants of the Wolffian duct (mesonephric duct). Malignant WAT occurring in the ovary is exceptionally uncommon. This article presents a case of malignant WAT in the ovary, analyzing and discussing its histological features, diagnostic challenges, biological behavior, and treatment options in conjunction with relevant literature to enhance our understanding of this rare tumor.

**Case presentation:**

A 64-year-old woman presented with an 8-month history of persistent abdominal pain and distension. An exploratory laparotomy revealed a small amount of pale-yellow ascites, a slightly atrophic uterus, and a left ovary without significant abnormalities. A solid mass measuring approximately 12 × 10 cm was observed between the left fallopian tube and ovary, displaying extensive dense adhesions to the posterior broad ligament and surrounding bowel. Frozen section pathology indicated a malignant tumor with necrotic areas suggestive of poorly differentiated carcinoma. The patient subsequently underwent a total hysterectomy, bilateral adnexectomy, omentectomy, pelvic lymphadenectomy, and pelvic adhesion release. Adjuvant chemotherapy with four cycles of paclitaxel and carboplatin (TC regimen) was administered, achieving normalization of tumor markers by the second cycle.

**Conclusions:**

WAT is a rare entity within the spectrum of female reproductive system tumors, predominantly benign in nature. Due to its extremely low incidence, standardized treatment protocols remain elusive. Further research is warranted to establish effective management strategies and provide a reference for future cases.

## Background

Wolffian adnexal tumor (WAT) is a rare neoplasm of the female reproductive system, with only a limited number of reported cases worldwide. It is believed to originate from mesonephric duct remnants, mostly found in the broad ligament, although cases have also been reported in the mesosalpinx, ovary, and retroperitoneum. Histopathological evaluation, including light microscopy, electron microscopy, and immunohistochemical analysis, reveals distinct features that differentiate WAT from Mullerian-origin tumors. First described by Kariminejad and Scully in 1973 as female adnexal tumors of probable Wolffian origin (FATWO) (1), WAT was later officially classified as a mesonephric duct remnant tumor in the WHO Classification of Tumours of Female Reproductive Organs (2). Although traditionally considered benign, malignant transformation of WAT is rare, yet some case reports have documented recurrence and metastasis (3). Due to the limited understanding of its biological behavior, further research is essential to establish standardized diagnostic and therapeutic strategies. Here, we report a case of malignant WAT with high proliferative activity, highlighting its histopathological and molecular characteristics, as well as its clinical management and follow-up outcomes.

## Case presentation

**Figure 1:**
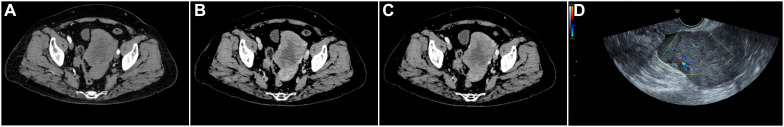
Imaging findings of an adnexal mass in a 64-year-old female patient. **(A–C)** Contrast-enhanced CT images reveal a well-defined soft-tissue mass (93.1 mm × 58.4 mm) in the left adnexal region, anterior to the uterus, with peripheral enhancement, a non-enhancing center, and mild dilation of the left ovarian vein. No pelvic or iliac lymphadenopathy is noted. **(D)** Gynecological ultrasound shows a hypoechoic, irregular mass (8.4 cm × 7.9 cm × 6.8 cm) in the left posterior uterine region, adjacent to a 1.7 cm × 1.2 cm structure resembling the left ovary, with moderate internal blood flow signals.

A 64-year-old female patient presented with an 8-month history of abdominal pain and distension. Contrast-enhanced CT revealed a well-demarcated, soft-tissue mass in the left adnexal region, anterior to the uterus, measuring approximately 93.1 mm × 58.4 mm. The lesion demonstrated prominent peripheral enhancement with a non-enhanced center and mild dilation of the left ovarian vein. No enlarged lymph nodes were observed in the pelvis or around the bilateral iliac vessels (**Figures 1A–C**). Gynecological examination detected a mobile mass, approximately 9 cm in diameter, in the anterior uterine region. Gynecological ultrasound showed a hypoechoic, irregularly shaped mass measuring 8.4 cm × 7.9 cm × 6.8 cm in the left posterior region of the uterus, adjacent to a 1.7 cm × 1.2 cm structure resembling the left ovary, with indistinct borders between them and moderately increased internal blood flow signals (**Figure 1D**). Tumor marker analysis revealed cancer antigen 125 (CA125) at 38.6 U/mL, human epididymis protein 4 (HE4) at 83.8 pmol/L, and neuron-specific enolase (NSE) at 30.8 ng/mL. Exploratory laparotomy identified a small amount of pale-yellow ascites, a slightly smaller uterus, and a 12 cm × 10 cm mass between the left fallopian tube and ovary, with dense adhesions to the posterior broad ligament and surrounding bowel. The left ovary appeared unremarkable, while the right adnexa and omentum were normal in appearance with no visible nodules. The liver and spleen surfaces were smooth, with no detectable nodules. Frozen pathological examination of the mass revealed features of a poorly differentiated malignant tumor with necrosis, raising the possibility of germ cell tumors, sex cord-stromal tumors, or metastatic carcinoma. Given the ambiguous findings and the potential for microscopic lymph node metastasis, systematic pelvic lymphadenectomy was performed to ensure accurate staging and comprehensive tumor clearance. Consequently, total hysterectomy, bilateral salpingo-oophorectomy, omentectomy, pelvic lymphadenectomy, and adhesion release were performed.

**Figure 2:**
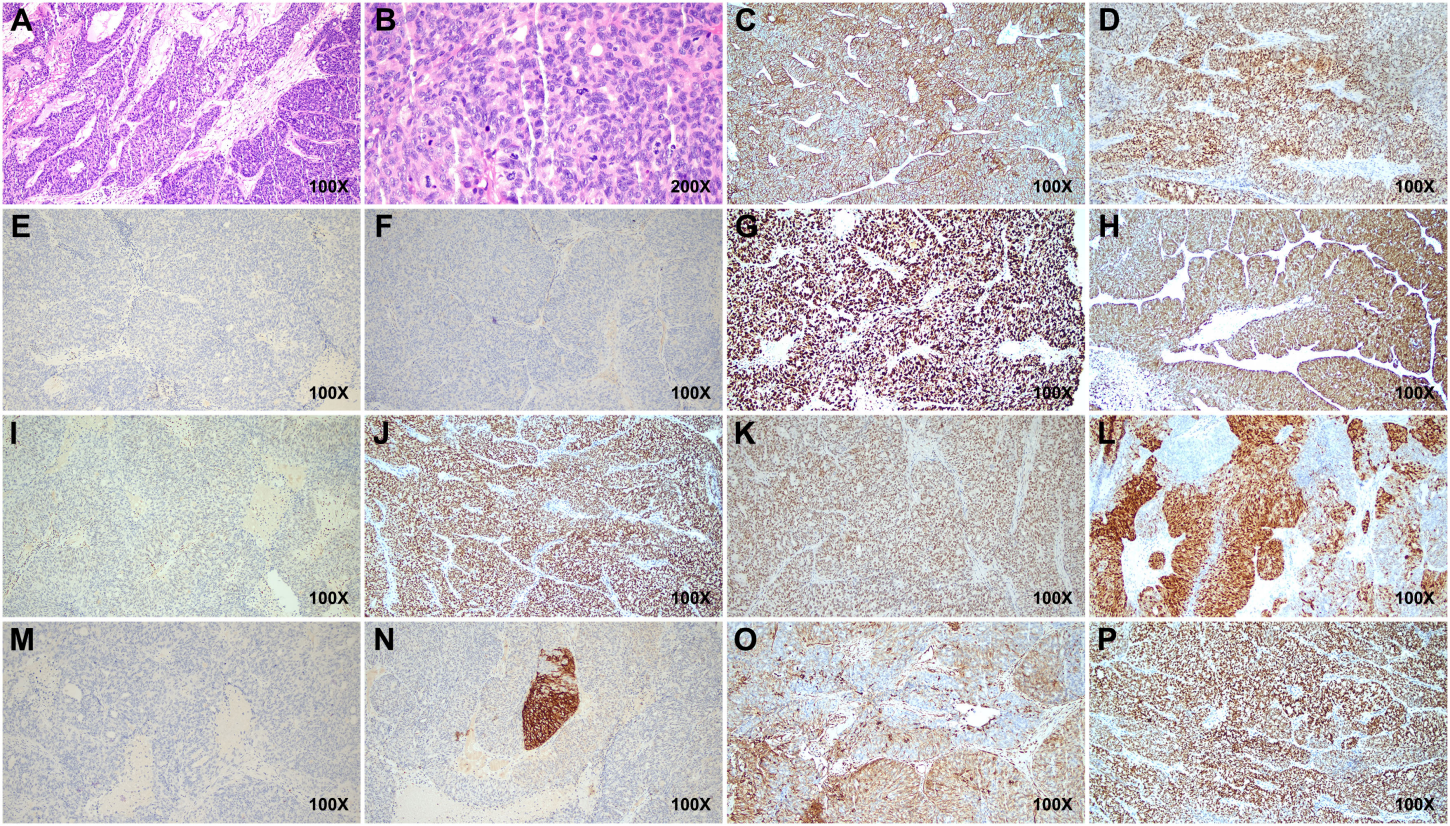
Histopathological and immunohistochemical features of the tumor. **(A, B)** Hematoxylin and eosin (H&E)-stained sections reveal tumor cells arranged in nests and trabeculae, showing marked nuclear atypia and frequent mitotic figures. **(C–P)** Immunohistochemical staining shows positive expressions for CK, ER, Ki-67, P16, Pax-8, PMS2, PR, Syn, Vimentin, and WT-1, while GATA3, Inhibin, and SALL4 are negative. P53 exhibits wild-type positivity. Scale bars indicate magnification. CK, creatine kinase; ER, estrogen receptor; Pax-8, paired box 8; Syn, synapsin.

**Figure 3:**
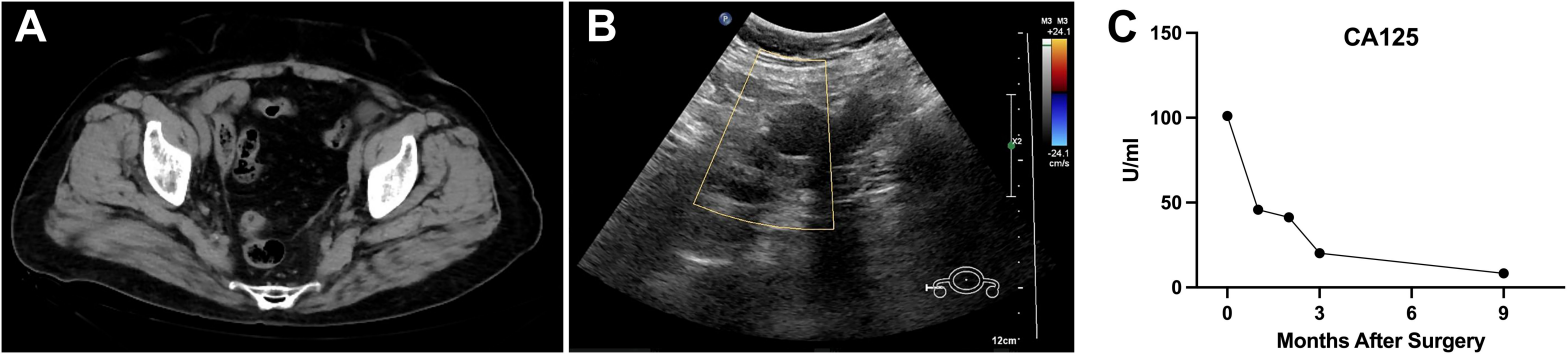
Postoperative follow-up imaging and tumor marker trends indicating no recurrence or metastasis. **(A, B)** Postoperative follow-up pelvic CT and gynecological ultrasound performed 9 months after surgery revealed no signs of recurrence or metastasis in the pelvic region. **(C)** Serial measurements of the tumor marker CA125 demonstrated a continuous downward trend post-surgery. CA125, cancer antigen 125.

Postoperative pathology revealed a poorly differentiated malignant tumor in the left adnexa (11 cm × 7 cm × 7 cm) with necrosis. Tumor cells were arranged in nests or trabeculae, exhibiting marked nuclear atypia and frequent mitotic figures (**Figures 2A, B**). Immunohistochemical analysis showed creatine kinase (CK) (+), estrogen receptor (ER) (+), GATA3 (−), Inhibin (−), Ki-67 (+), P16 (+), P53 (+, wild type), paired box 8 (Pax-8) (+), PMS2 (+), Progesterone receptor (PR) (++), SALL4 (−), synapsin (Syn) (+), Vimentin (focal +), and WT-1 (+), indicating an embryonic origin of the tumor (**Figures 2C–P**). Genetic testing confirmed a Breast cancer type 1 (BRCA1) systemic mutation with established clinical significance and a TP53 mutation with potential clinical relevance. The patient was diagnosed with Wolffian adnexal tumor (International Federation of Gynecology and Obstetrics (FIGO) stage IC) and underwent four cycles of adjuvant chemotherapy with paclitaxel and carboplatin (TC regimen). The patient has been regularly followed up post-treatment. At 9 months after surgery, pelvic CT and gynecological ultrasound revealed no signs of recurrence or metastasis (**Figures 3A, B**). Additionally, serial tumor marker assessments demonstrated a sustained decline in CA125 levels (**Figure 3C**).

## Discussion and conclusion

WAT is an exceptionally rare entity, with fewer than 90 cases reported in the literature to date (4). The age of onset ranges widely from 15 to 83 years, with a mean age of approximately 50 (5). WAT typically presents without specific clinical symptoms and is often an incidental finding during surgery (6). In some cases, patients may experience non-specific symptoms such as abdominal pain, distension, or an abdominal mass and may experience pressure symptoms like vaginal bleeding, urinary frequency, or constipation. WATs are most often found as unilateral adnexal masses, frequently on the right side (7). While specific tumor markers are lacking, some cases may show elevated CA125 and estradiol levels, with histopathological examination remaining the definitive diagnostic standard (8, 9).

Currently, there is no consensus on the diagnostic criteria for malignant WAT. WATs tend to arise in specific locations, including the broad ligament, mesosalpinx, fallopian tube, ovary, and retroperitoneum, where mesonephric duct remnants may be found. Tumor sizes vary considerably, from 0.8 to 25 cm (4, 10). Grossly, WATs are encapsulated and well-demarcated, with gray-yellow or light-brown cut surfaces, and often have a solid, occasionally nodular or cystic appearance with focal hemorrhage or cystic necrosis. Microscopically, WAT cells typically exhibit mild atypia and cribriform or glandular patterns and rarely show mitotic figures (11). Immunohistochemical markers play a pivotal role in diagnosing WAT, distinguishing it from other pelvic tumors. WAT typically expresses CK, PAX8, and GATA3, markers indicative of Wolffian duct origin. TTF-1 and Vimentin may also be positive, while CD10 and α-inhibin are inconsistently expressed (12, 13). Negative or weak expression of ER and PR further differentiates WAT from endometrial and sex cord-stromal tumors (14–16). In this case, the tumor exhibited CK (+), PAX8 (+), WT1 (+), and focal expression of Vimentin, supporting its Wolffian origin. The absence of GATA3, Inhibin, and SALL4 ruled out mesonephric carcinoma and germ cell tumors. Additionally, the presence of wild-type P53 and a Ki-67 index above 30% highlighted the tumor’s malignant potential.

The criteria proposed by Sivridis, including tumor size (>100 mm), hypercellularity, capsular invasion, and tumor implants, are often applied in clinical studies to guide diagnosis (17). A systematic diagnostic approach integrating morphology and Immunohistochemistry (IHC) findings is crucial for differentiating WAT, particularly malignant variants. Key indicators of malignancy include necrosis, capsular invasion, and increased mitotic activity. Differential diagnosis of pelvic masses is particularly challenging in cases like this, and the unique morphological and immunohistochemical profiles of WAT play a crucial role in distinguishing it from other neoplasms. Sertoli stromal tumors may exhibit tubular structures similar to WAT; however, WAT is characterized by solid spindle-cell areas and cystic structures. Additionally, Sertoli stromal tumors primarily arise in the ovary and are often associated with endocrine symptoms, whereas WAT typically originates from mesonephric duct remnants in extra-ovarian locations, such as the broad ligament and mesosalpinx (18). Granulosa cell tumors also commonly originate in the ovary and display coffee bean-like nuclei with Call–Exner bodies, features absent in WAT. Furthermore, Periodic Acid Schiff (PAS) staining of granulosa cell tumors reveals peritubular basement membrane structures, whereas WAT lacks these findings (19). Endometrioid adenocarcinoma exhibits greater cellular atypia, a high mitotic index, and frequent squamous metaplasia. Immunohistochemically, it is positive for Epithelial membrane antigen (EMA), PR, and ER, with a high Ki-67 index, whereas WAT typically lacks these markers or exhibits lower expression (20). Serous papillary adenocarcinoma is often cystic and fragile, with complex papillary structures and psammoma bodies. It expresses CKpan (AE1/3), CK7, EMA, and WT1, whereas WAT lacks α-inhibin expression, further aiding differentiation (21).

The diagnostic accuracy for malignant WAT is reported to be as low as 17% (22). Histopathological features indicative of malignancy include intratumoral necrosis, capsular invasion, high mitotic activity, and marked cellular atypia (8). Malignant WAT has an estimated recurrence rate of 11%, with a median interval of 48 months, primarily affecting the lungs and liver (22, 23). Given the rarity of malignant WAT and the limited prognostic value of morphological and immunohistochemical markers, long-term follow-up is essential, yet no standardized treatment guidelines exist (24). Existing case reports often extrapolate treatment strategies from malignancies of similar anatomical origin. Prognosis is primarily determined by clinicopathologic staging, cellular atypia, and proliferation indices (3). Complete surgical resection, including hysterectomy, bilateral adnexectomy, and cytoreductive surgery, remains the preferred primary treatment for malignant WAT (6). In the present case, due to the poorly differentiated nature of the tumor, systematic lymphadenectomy was performed despite the absence of intraoperative evidence of lymph node involvement. This approach ensured accurate staging and ruled out microscopic nodal metastases, aligning with oncological principles for malignancies with uncertain biological behavior. The role of chemotherapy and radiotherapy remains controversial. Subsequently, the patient received four cycles of paclitaxel and carboplatin (TC regimen). Regular follow-up post-treatment revealed no evidence of recurrence or metastasis at 9 months, as confirmed by pelvic CT and gynecological ultrasound (**Figures 3A, B**). Additionally, serial CA125 measurements demonstrated a continuous decline post-surgery, further supporting the absence of residual or recurrent disease (**Figure 3C**). Long-term follow-up should be conducted to continuously monitor disease status, allowing for the timely detection and management of any recurrence or metastasis.

The molecular characteristics of WAT remain incompletely defined; however, they may provide valuable diagnostic and therapeutic insights. In our case, the presence of a BRCA1 mutation suggests a potential hereditary predisposition. Further investigation of molecular markers, including c-KIT and TP53, may improve diagnostic accuracy and facilitate personalized treatment strategies. Integrating immunohistochemical profiling with molecular analysis offers a comprehensive approach to WAT diagnosis. Currently, Poly (ADP-ribose) Polymerase (PARP) inhibitors are recommended for patients with BRCA mutations in advanced epithelial ovarian cancer (25), yet their efficacy in WAT remains unexplored. In contrast, targeted therapies for TP53 mutations are still under clinical investigation. Notably, Steed et al. (26) reported a case of malignant WAT in a 15-year-old female patient who was refractory to surgery and chemotherapy but responded to Gleevec, a tyrosine kinase inhibitor targeting CD117 (27, 28). These findings suggest that molecular-targeted therapies based on mutations such as c-KIT, BRCA, and TP53 could represent a promising treatment avenue for malignant WAT, warranting further research to validate their efficacy (29, 30).

In conclusion, WAT presents significant diagnostic and therapeutic challenges due to its rarity, non-specific clinical manifestations, and the absence of standardized diagnostic criteria. A multidisciplinary approach integrating comprehensive morphological assessment, immunohistochemical profiling, and molecular analysis is crucial for accurate diagnosis and differentiation from other pelvic tumors. Advances in elucidating the molecular characteristics of WAT, particularly mutations in genes such as BRCA1 and TP53, provide novel insights into its pathogenesis and potential therapeutic targets. Given the limited number of reported cases, long-term follow-up and further research are essential to establish evidence-based management strategies and to evaluate the efficacy of molecular-targeted therapies in improving clinical outcomes.

## Data Availability

The original contributions presented in the study are included in the article/supplementary material. Further inquiries can be directed to the corresponding author.
